# Association of Serum IL-35 and TGF-β Levels with the Incidence, Severity, and Treatment Response in Iraqi Patients with Graves’ Disease

**DOI:** 10.30699/ijp.2025.2066164.3489

**Published:** 2025-08-15

**Authors:** Zahraa L. Hussein, Karrar S. Zayed

**Affiliations:** Department of Laboratory Investigations, Faculty of Science, University of Kufa, Najaf, Iraq

**Keywords:** Graves’ disease, IL-35, TGF-β, thyrotropin receptor antibody, carbimazole

## Abstract

**Background & Objective:**

Graves’ disease (GD) is a common autoimmune thyroid disorder characterized by thyroid hormone hypersecretion and autoantibody formation against the thyrotropin receptor (TRAb). Interleukin-35 (IL-35), a novel immunosuppressive cytokine of the IL-12 family secreted primarily by regulatory T and B cells, has been implicated in the modulation of autoimmune responses. Transforming growth factor β (TGF-β) is a cytokine with immunoregulatory properties that plays a central role in T-regulatory cell differentiation. This study aimed to investigate serum IL-35 and TGF-β levels in Iraqi patients with GD and assess their association with disease severity, clinical manifestations, and response to carbimazole therapy.

**Methods:**

A total of 103 participants were enrolled, including 66 patients with GD (mean age, 41.6 [SD, 13.95] years) and 37 healthy controls (mean age, 37.43 [SD, 10.25] years). Serum IL-35 and TGF-β concentrations were measured using enzyme-linked immunosorbent assay (ELISA).

**Results:**

IL-35 levels were significantly reduced in untreated GD patients compared with healthy controls and partially increased after carbimazole therapy (*P < .001*). TGF-β levels were highest in untreated patients and declined after treatment (*P < .001*). IL-35 showed no correlation with FT3, FT4, or TRAb but was elevated in patients with disease duration longer than 5 years (*P = .046*) and in those with severe exophthalmos (*P = .03*). TGF-β levels were inversely correlated with FT3, FT4, and TRAb (*P < .05*) and were higher in carbimazole nonresponders and in patients with severe exophthalmos (*P < .05*).

**Conclusion:**

IL-35 may serve as a promising biomarker for monitoring disease activity, prognosis, and therapeutic response in patients with GD.

## Introduction

Graves’ disease (GD) is an autoimmune disorder of the thyroid gland that occurs most frequently in individuals with a familial predisposition. It is the leading cause of hyperthyroidism and is more common in women. A key feature of GD is the production of autoantibodies against the thyrotropin receptor (TRAb) (1-3). The hypothalamic hormone thyrotropin-releasing hormone (TRH) stimulates secretion of thyroid-stimulating hormone (TSH), which in turn promotes the thyroid gland to release free triiodothyronine (FT3) and free thyroxine (FT4), the primary regulators of metabolic activity (4). GD develops as a consequence of immune tolerance breakdown to organ-specific self-antigens, primarily the TSH receptor. Measurement of TSH levels provides a useful diagnostic and monitoring tool in patients with GD, both before and during treatment (5).

Interleukin 35 (IL-35) is a heterodimeric cytokine of the IL-12 family, secreted mainly by regulatory T cells (Tregs) and regulatory B cells (Bregs). It exerts potent anti-inflammatory effects by suppressing effector T-cell proliferation and promoting Treg expansion, thereby contributing to the maintenance of immune tolerance (6). Altered IL-35 expression has been reported in several autoimmune diseases, including systemic lupus erythematosus (SLE), inflammatory bowel disease (IBD), and Hashimoto thyroiditis (HT) (7-9). Although IL-35 has been recognized as an important immunoregulatory cytokine, its role in GD remains unclear. Previous findings are inconsistent; some studies reported significantly reduced IL-35 levels in autoimmune thyroid disease compared with healthy individuals (10,11), whereas others demonstrated increased IL-35 concentrations in GD, with potential links to immune cell dynamics and disease severity (12,13).

Transforming growth factor β (TGF-β), a multifunctional cytokine of the TGF-β superfamily, is secreted by a wide range of immune and nonimmune cells. It regulates cell proliferation, differentiation, apoptosis, and immune tolerance (14). Elevated serum TGF-β concentrations have been observed in GD patients in several studies (15-17). Conversely, reduced TGF-β levels have been associated with impaired peripheral tolerance and increased autoimmune reactivity, suggesting a possible contribution to GD pathogenesis (18).

Given these conflicting data, the present study was designed to evaluate serum IL-35 and TGF-β concentrations in Iraqi patients with GD and to determine their associations with disease activity, response to carbimazole therapy, and selected clinical features.

## Materials and Methods

### Patients and Clinical Samples

This case-control study included 103 participants: 66 patients with GD and 37 healthy controls. Among the patients, 21 were newly diagnosed and untreated, while 45 were receiving carbimazole therapy. All subjects were recruited from the Diabetes and Endocrinology Center at Al-Sadr Medical City, Najaf, Iraq.

Inclusion criteria were clinical diagnosis of GD, elevated thyroid hormone levels, and positive TRAb test results. Exclusion criteria were coexisting autoimmune disorders (eg, Hashimoto thyroiditis, systemic lupus erythematosus), infections, malignancy, chronic kidney or liver disease, cardiovascular disease, psychiatric illness, diabetes, pregnancy, or prior radioiodine therapy.

From each participant, 5 mL of venous blood was collected under aseptic conditions. Samples were left at room temperature for 30–60 minutes to clot, then centrifuged to obtain serum. The serum was aliquoted and stored at −20 °C until analysis. The study was conducted in accordance with the Declaration of Helsinki and approved by the Ethics Committee of the Faculty of Science, Kufa University.

### Estimation of IL-35, TGF-β, TRAb, TSH, FT3, and FT4 Levels

Serum IL-35, TGF-β, and TRAb concentrations were measured using enzyme-linked immunosorbent assay (ELISA) kits (BT LAB, China) based on the double-antibody sandwich principle. All procedures were performed according to the manufacturer’s instructions. Serum samples were diluted 1:2 to ensure values fell within the linear range of the standard curve. Each sample was tested in duplicate. Plates were incubated at 37 °C for 60 minutes with three washes between steps. Tetramethylbenzidine (TMB) substrate was used for color development, and absorbance was measured at 450 nm with a Bio-Rad iMark microplate reader. Concentrations were calculated from the standard curve provided with the kit.

TSH, FT3, and FT4 concentrations were measured using electrochemiluminescence immunoassay (ECLIA) on an automated analyzer (Cobas e411, Roche Diagnostics, Switzerland).

### Statistical Analysis

Data were analyzed using SPSS version 25.0 (IBM Corp). Continuous variables were expressed as mean ± standard deviation (SD), and categorical variables as frequencies and percentages. Group comparisons were performed using independent-sample t test or ANOVA, as appropriate. Pearson correlation coefficients were calculated to assess relationships between IL-35, TGF-β, and clinical or biochemical variables. A 2-tailed *P* value less than .05 was considered statistically significant.

## Results

### Serum IL-35 and TGF-β Levels Among the Study Groups

Serum IL-35 levels differed significantly among the three groups (*P < .001*). The lowest concentrations were observed in untreated GD patients (4.94 ± 1.15 pg/mL), followed by treated patients (6.23 ± 5.01 pg/mL), whereas the highest levels were recorded in healthy controls (12.77 ± 14.20 pg/mL) (Table 1). Similarly, serum TGF-β concentrations varied significantly across the groups (*P < .001*). Untreated GD patients showed the highest levels (156.81 ± 88.18 pg/mL), followed by treated patients (96.45 ± 81.60 pg/mL), while the lowest levels were detected in the control group (74.46 ± 57.47 pg/mL) (Table 2).

### Clinical and Sociodemographic Characteristics in GD Patients

Neither IL-35 nor TGF-β levels were significantly associated with sex, age, smoking status, iodine intake, family history of thyroid disease, or disease severity (*P > .05*). However, TGF-β concentrations were significantly associated with treatment response (*P < .05*), whereas IL-35 levels were not (*P > .05*) (Table 3).

IL-35 levels were significantly higher in patients with disease duration longer than 5 years (6.51 ± 6.18 pg/mL) compared with those with shorter duration (4.90 ± 1.09 pg/mL; *P = .046*). No significant difference in TGF-β concentrations was observed based on disease duration (*P = .39*). Both IL-35 and TGF-β levels were elevated in patients with severe exophthalmos (7.42 ± 7.68 pg/mL and 177.65 ± 93.92 pg/mL, respectively), with statistically significant differences for IL-35 (*P = .03*) and borderline significance for TGF-β (*P = .05*).

### Correlation of IL-35 and TGF-β with Thyroid Parameters and TRAb in GD Patients

No significant correlations were identified between IL-35 concentrations and TSH, FT3, FT4, or TRAb (*P > .05*) (Table 4). In contrast, TGF-β concentrations showed significant inverse correlations with FT3 (*r* = –0.437, *P < .001*), FT4 (*r* = –0.339, *P = .005*), and TRAb (*r* = –0.244, *P = .049*). The correlation with TSH was not significant (*P = .054*). Strong positive correlations were found between FT3 and FT4 (*r* = 0.887, *P < .001*) and between each of these hormones and TRAb (*r* = 0.587 and *r* = 0.654, respectively; *P < .001*) (Table 5).

**Table 1:** Serum IL-35 levels in healthy controls and in patients with Graves’ disease (GD) treated and untreated with carbimazole

Group	N	Mean (pg/mL)	SD	95% CI for Mean	*P* value
Healthy controls	37	12.77	14.20	8.03 – 17.50	*P < .001*
GD, treated with carbimazole	45	6.23	5.01	3.94 – 8.51	
GD, untreated	21	4.94	1.15	4.60 – 5.29	

**Table 2:** Serum TGF-β levels in healthy controls and in patients with Graves’ disease (GD) treated and untreated with carbimazole

Group	N	Mean (pg/mL)	SD	95% CI for Mean	*P* value
Healthy controls	37	74.46	57.47	55.30 – 93.63	*P < .001*
GD, treated with carbimazole	45	96.45	81.60	72.32 – 120.57	
GD, untreated	21	156.81	88.18	115.31 – 198.30	

**Table 3:** Serum IL-35 and TGF-β levels according to clinical and sociodemographic characteristics in patients with Graves’ disease (GD)

Characteristic	N	IL-35, Mean ± SD (pg/mL)	*P* value	TGF-β, Mean ± SD (pg/mL)	*P* value
Sex					
Male	21	4.94 ± 1.23	.44	146.53 ± 94.93	.58
Female	45	5.54 ± 3.53		133.44 ± 88.52	
Age					
≤ 40 y	33	5.93 ± 4.08	.115	122.01 ± 91.85	.16
> 40 y	33	4.77 ± 0.94		153.20 ± 86.87	
Disease duration					
≤ 5 y	50	4.94 ± 1.09	.046*	142.99 ± 95.71	.39
> 5 y	16	6.65 ± 5.71		120.79 ± 69.58	
Family history					
Yes	16	5.44 ± 2.17	.89	158.44 ± 118.76	.29
No	50	5.32 ± 3.23		130.94 ± 79.12	
Smoking					
Yes	8	5.07 ± 0.79	.78	184.90 ± 79.19	.11
No	58	5.39 ± 3.19		131.08 ± 90.15	
Iodine treatment					
Yes	12	4.84 ± 0.74	.51	100.39 ± 85.65	.114
No	54	5.46 ± 3.29		145.88 ± 89.70	
Response to carbimazole					
Responders	26	5.32 ± 2.29	.16	132.19 ± 100.87	.04*
Nonresponders	19	4.52 ± 1.06		185.23 ± 65.59	
Exophthalmos					
Simple	39	5.43 ± 3.17	.03*	171.14 ± 88.45	.050
Severe	5	10.03 ± 9.30		177.65 ± 93.92	
Asymptomatic	22	5.45 ± 2.24		117.91 ± 85.06	
Disease severity					
Mild	40	5.26 ± 2.07	.56	88.22 ± 64.74	.165
Moderate	17	5.74 ± 4.17		142.37 ± 94.83	
Severe	9	6.94 ± 4.62		150.94 ± 89.95	

**Table 4:** Correlations of IL35 level with TSH, FT3, FT4 and TRAb levels in GD patients

		**IL** **-** **35**	**TSH**	**FT3**	**FT4**	**TRA** **b**
IL-35	Pearson Correlation	1	-0.095	0.210	0.062	0.002
	*p.* value		0.447	0.090	0.620	0.988
	N	66	66	66	66	66
TSH	Pearson Correlation	-0.095	1	-0.193	-0.184	-0.001
	*p.* value	0.447		0.121	0.140	0.996
	N	66	66	66	66	66
FT3	Pearson Correlation	0.210	-0.193	1	0.887^**^	0.587^**^
	*p.* value	0.090	0.121		0.000	0.000
	N	66	66	66	66	66
FT4	Pearson Correlation	0.062	-0.184	0.887^**^	1	0.654^**^
	*p.* value	0.620	0.140	0.000		0.000
	N	66	66	66	66	66
TRAb	Pearson Correlation	0.002	-0.001	0.587^**^	0.654^**^	1
	*p.* value	0.988	0.996	0.000	0.000	
	N	66	66	66	66	66

**Table 5:** Correlations of TGF-β level with TSH, FT3, FT4, and TRAb levels in GD patients

		**TSH**	**FT3**	**FT4**	**TRA** **b**	**TGF-β**
TSH	Pearson Correlation	1	-0.193	-0.184	-0.001	0.238
	*p.* value		0.121	0.140	0.996	0.054
	N	66	66	66	66	66
FT3	Pearson Correlation	-0.193	1	0.887^**^	0.587^**^	-0.437^**^
	*p.* value	.121		0.0001	0.0001	0.0001
	N	66	66	66	66	66
FT4	Pearson Correlation	-0.184	0.887^**^	1	0.654^**^	-0.339^**^
	*p.* value	0.140	0.0001		0.0001	0.005
	N	66	66	66	66	66
TRAb	Pearson Correlation	-0.001	0.587^**^	0.654^**^	1	-0.244^*^
	*p.* value	0.996	0.0001	0.0001		0.049
	N	66	66	66	66	66
TGF-β	Pearson Correlation	0.238	-0.437^**^	-0.339^**^	-0.244^*^	1
	*p.* value	0.054	0.000	0.005	0.049	
	N	66	66	66	66	66

## Discussion

This study demonstrated a significant decrease in IL-35 levels in untreated Graves’ disease (GD) patients, suggesting that suppression of this anti-inflammatory cytokine contributes to the immune imbalance underlying the disease. Following carbimazole therapy, IL-35 levels partially increased, indicating that treatment exerts an immunomodulatory effect beyond inhibition of thyroid hormone synthesis. These findings imply that low IL-35 is not merely a consequence of GD but may play a causal role in its progression, while its partial restoration could serve as a marker of therapeutic response. To our knowledge, this is the first Iraqi study to measure serum IL-35 levels in GD patients both before and after treatment.

The reduction in IL-35 observed in untreated GD aligns with the disrupted Treg function and elevated TRAb that foster a Th1/Th17-skewed inflammatory environment, promoting chronic tissue damage (19). Consistent with our results, decreased IL-35 levels have been reported in other autoimmune diseases, including Hashimoto thyroiditis, systemic lupus erythematosus, and inflammatory bowel disease, suggesting a common immunopathological mechanism (8–12). However, contrasting reports exist. A Chinese cohort described increased IL-35 levels in GD, highlighting that IL-35 dynamics may vary by disease stage, ethnicity, or methodological differences (21). Such discrepancies may reflect phase-dependent fluctuations: reduced IL-35 during active disease due to impaired Treg-derived production, versus later elevations representing a compensatory yet insufficient immune response (12). Genetic background, environmental factors, and assay variations may also explain these differences. Collectively, these findings strengthen the concept that IL-35 deficiency is characteristic of active GD and a potential biomarker of disease activity.

TGF-β dynamics in our study also provide important insights. Untreated GD patients had elevated serum TGF-β, likely reflecting a compensatory response to uncontrolled autoimmune activation (22). Treated patients showed intermediate levels, consistent with the immunosuppressive effects of carbimazole, which may attenuate pro-inflammatory signaling and thereby reduce TGF-β production (15, 23, 24). Other studies, however, have reported reduced TGF-β in GD, suggesting cytokine depletion or impaired Treg function in advanced disease (25). Even when elevated, TGF-β signaling may be functionally ineffective due to “TGF-β resistance,” wherein inflammatory cytokines such as IL-6 and TNF-α disrupt its pathway (26, 27). Notably, in the presence of IL-6, TGF-β may paradoxically promote Th17 differentiation rather than Treg expansion, thereby amplifying autoimmune responses.

We also observed that IL-35 levels correlated with disease duration and exophthalmos severity, but not with demographic or environmental factors. Elevated IL-35 in long-standing disease and severe orbitopathy may reflect localized or time-dependent immunomodulation, in line with previous studies showing functionally impaired IL-35⁺Bregs in thyroid-associated ophthalmopathy (28, 29). Similarly, higher TGF-β levels in non-responders to carbimazole suggest persistent immune activation, whereas lower levels in responders may reflect successful immunosuppression (15, 23). This is consistent with TGF-β’s known role in orbital fibrosis and inflammation (16). Yet, given conflicting data, TGF-β alone may not fully capture systemic disease activity, as chronic inflammation and cytokine-mediated resistance can impair its immunoregulatory function (25–27, 30).

Importantly, no significant correlation was detected between IL-35 and thyroid hormone levels or autoantibodies, consistent with some studies (19, 31, 32) but contrasting with an Iraqi report that found parallel decreases in IL-35 and TRAb (7, 27). This supports the notion that IL-35 regulation in GD occurs independently of thyroid hormone dynamics, reflecting complex immune-endocrine interactions beyond classical feedback loops (38–43). In contrast, TGF-β levels inversely correlated with FT3, FT4, and TRAb, reinforcing its role as part of an insufficient compensatory immune response (44-47).

Overall, these findings highlight the intricate interplay of IL-35 and TGF-β in GD pathogenesis. IL-35 deficiency appears central to disease activity, with partial restoration by carbimazole underscoring its potential as a biomarker of therapeutic response. TGF-β elevation, although compensatory, may be limited by resistance mechanisms that shift its role from immune regulation to inflammation. Future studies with larger cohorts and longitudinal follow-up are warranted to clarify cytokine dynamics across disease stages and treatment responses, and to evaluate their potential as biomarkers and therapeutic targets in GD.

## Conclusion

This study evaluated IL-35 and TGF-β as potential immunological markers in Graves’ disease. IL-35 levels were reduced in untreated patients and partially restored after carbimazole therapy, highlighting its possible role in immune regulation and treatment response. In contrast, TGF-β concentrations were elevated in untreated and nonresponsive cases, suggesting a compensatory but insufficient regulatory effect. Its inverse correlation with FT3, FT4, and TRAb further supports its involvement in autoimmune modulation. Collectively, these findings indicate that both IL-35 and TGF-β may serve as valuable biomarkers for disease activity and therapeutic outcomes in Graves’ disease.

## Data Availability

There is no additional data separate from available in cited references.
